# Diagnosis of 17-alpha hydroxylase deficiency performed late in life in a patient with a 46,XY karyotype

**DOI:** 10.1530/EDM-22-0338

**Published:** 2023-05-18

**Authors:** Bruno Bouça, Mariana Cascão, Pedro Fiúza, Sara Amaral, Paula Bogalho, José Silva-Nunes

**Affiliations:** 1 Department of Endocrinology, Diabetes and Metabolism - Centro Hospitalar Universitário de Lisboa Central, Lisbon, Portugal; 2 Nova Medical School/ Faculdade de Ciencias Medicas, Universidade Nova de Lisboa, Lisbon, Portugal; 3 Intensive Care Unit - Centro Hospitalar Universitário de Lisboa Central, Lisbon, Portugal; 4 Department of Internal Medicine, Unit 7.2 - Centro Hospitalar Universitário de Lisboa Central, Lisbon, Portugal; 5 Health and Technology Research Center (H&TRC), Escola Superior de Tecnologia da Saude de Lisboa, Lisbon, Portugal

**Keywords:** Adult, Female, Asian - other, Portugal, Adrenal, Adrenal, Unique/unexpected symptoms or presentations of a disease, May, 2023

## Abstract

**Summary:**

17-Alpha-hydroxylase deficiency (17OHD) is a rare autosomal recessive disease, representing 1% of cases of congenital adrenal hyperplasia. A 44-year-old female presented to the emergency department complaining of generalized asthenia and polyarthralgia for about 2 weeks. On examination, she was hypertensive (174/100 mmHg), and laboratory results revealed hypokalemia and hypocortisolism. She had an uncharacteristic morphotype, BMI of 16.7 kg/m^2^, cutaneous hyperpigmentation, and Tanner stage M1P1, with normal female external genitalia. She reported to have primary amenorrhea. Further analytical evaluations of her hormone levels were performed CT scan revealed adrenal bilateral hyperplasia and absence of female internal genitalia. A nodular lesion was observed in the left inguinal canal with 25 × 10 mm, compatible with a testicular remnant. Genetic analysis identified the c.3G>A p.(Met1?) variant in homozygosity in the CYP17A1 gene, classified as pathogenic, confirming the diagnosis of 17OHD. Karyotype analysis was compatible with 46,XY. The association of severe hypokalemia, hypertension, hypocortisolism, and oligo/amenorrhea and the absence of secondary sexual characteristics favored the diagnosis of 17OHD, confirmed by genetic testing. As in other published clinical cases, diagnosis outside pediatric age is not rare and should be considered when severe hypokalemia occurs in hypertensive adults with a lack of secondary sexual characteristics.

**Learning points:**

- The association of severe hypokalemia, hypertension, hypocortisolism, and oligo/amenorrhea and the absence of secondary sexual characteristics favor the diagnosis of 17-alpha-hydroxylase deficiency (17OHD).
- Diagnosis outside pediatric age is not rare.
- 17OHD should be considered when severe hypokalemia occurs in hypertensive adults with a lack of secondary sexual characteristics.

## Background

*CYP17A1* gene mapped at chromosome 10q encodes 17-hydroxylation enzyme and 17,20-lyase. More than 100 mutations have been described in the *CYP17A1* gene (1, 2). Most of them are a single-based substitution that changes amino acids or introduces a stop codon into the mRNA transcript, but deletions, conversions, and mutations affecting the donor site splicing have also been described (3).

17-alpha-hydroxylase deficiency (17OHD) is a rare autosomal recessive disorder caused by mutations in the *CYP17A1* gene, with an estimated incidence of 1 in 50 000, accounting for only 1% of congenital adrenal hyperplasia (CAH) (4). It causes a decrease in cortisol, androgen, and estrogen production with a subsequent increase in adrenocorticotrophic hormone (ACTH) and gonadotropin levels (5). High ACTH levels contribute to increased production and accumulation of 17-deoxycorticosteroids, especially deoxycorticosterone. Excessive levels of mineralocorticoids lead to volume expansion, hypertension, hypokalemia, and high, normal, or suppressed aldosterone, with oligo/amenorrhea in females and pseudo-hermaphroditism in males (6). Despite a decline in cortisol levels, this deficiency does not manifest as classical adrenal insufficiency due to increased corticosterone production, which exerts a mild glucocorticoid effect and plays a negative feedback effect on ACTH secretion at the pituitary (7, 8, 9). Androgen and estrogen production is also affected because 17-hydroxylase/17,20-lyase is also present in the gonads, playing a key role in sexual maturity throughout fetal life and puberty (10). This results in ambiguous or female external genitalia in affected male individuals and absent or delayed pubertal development in female patients (11).

## Case presentation

A 44-year-old woman, diagnosed with hypertension since 20 years old and without any chronic medication, presented at the emergency department, in February 2020, complaining of generalized asthenia and polyarthralgia for about 2 weeks. She was hypertensive (174/100 mmHg), with a heart rate of 70 bpm, eupneic, and apyretic on examination. Laboratory results revealed the following: acute kidney lesion – Cr: 1.39 mg/dL (normal range (NR): 0.57–1.11); severe hypokalemia – K+: 1.2 mEq/L (NR: 3.5–5.1); rhabdomyolysis – creatine kinase (CK): 9934 U/L (NR: 29–168); and respiratory alkalosis – pH: 7.56 (NR: 7.35–7.45), HCO_3_: 18.3 mmol/L (NR: 22–26), pCO_2_: 24 mmHg (NR: 35–45). ECG showed ST-segment deviations in DI, DII, and V3–V6. Thoracic x-ray showed an increased cardiothoracic index.

After starting ionic correction with potassium, she had a syncope episode with electrocardiographic tracing of ventricular tachycardia, followed by ‘Torsades de Pointes’ and subsequent asystole. Reanimation maneuvers were performed with the administration of two shocks (200 + 360 J) with conversion to sinus rhythm. She was then transferred to the intensive care unit (ICU), with rapid clinical improvement under antihypertensive therapy and hydrocortisone i.v., while hypokalemia remained despite i.v. correction. Further laboratory evaluation showed: the following cortisol <0.4 µg/dL (NR: 3.7–19.4), ACTH 213 pg/mL (NR: <46), aldosterone (decubitus) 27.4 ng/dL (NR: 1–16), and renin <1.8 µUI/mL (NR: 2.8–39.9). Due to these findings, the ICU team contacted the endocrinology department to manage the patient’s etiological study and transferred the patient to our ward.

When questioned, the patient said there was no family history of chronic illness, including hypertension. Both her father (height of 169 cm) and her mother (height of 165 cm) died of lung disease and were not consanguineous. She reported having had no menarche and never being pregnant. She had an older sister, height 165 cm, with normal female phenotype and secondary sexual development, with regular menstruation and three children.

## Investigation

**Figure 1:**
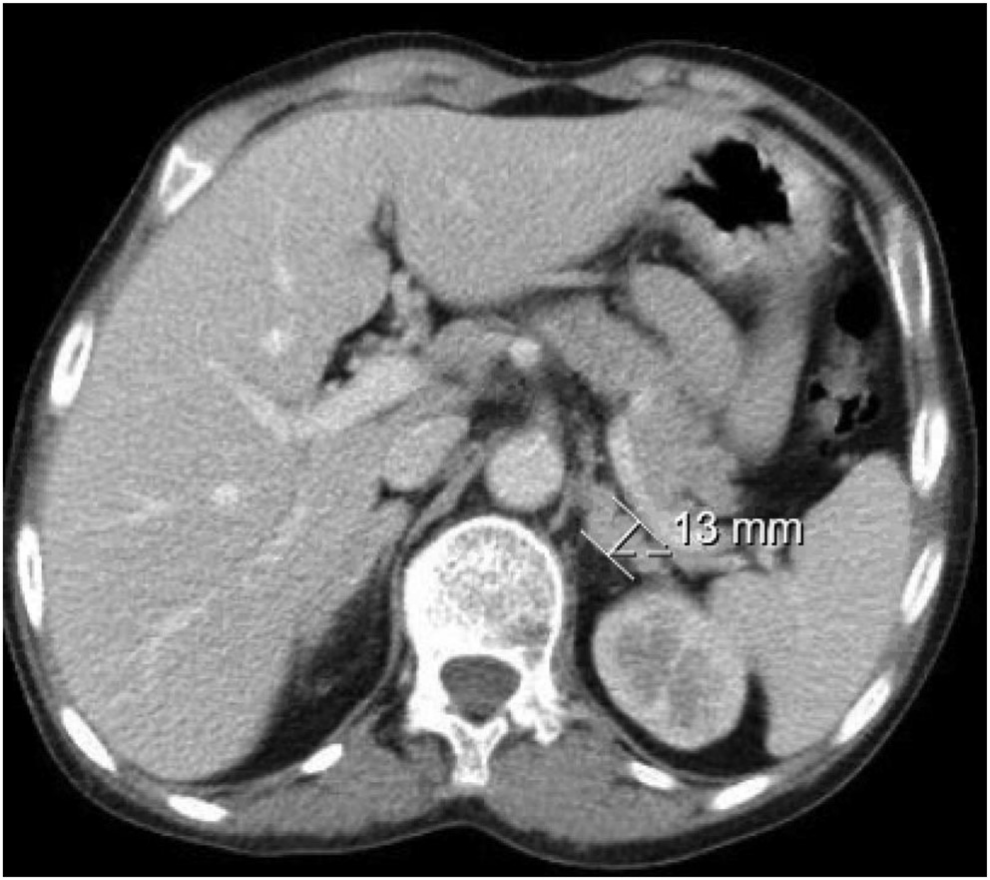
Abdominal CT scan showing adrenal bilateral hyperplasia – right width 11.5 mm, left width 12.9 mm.

**Figure 2:**
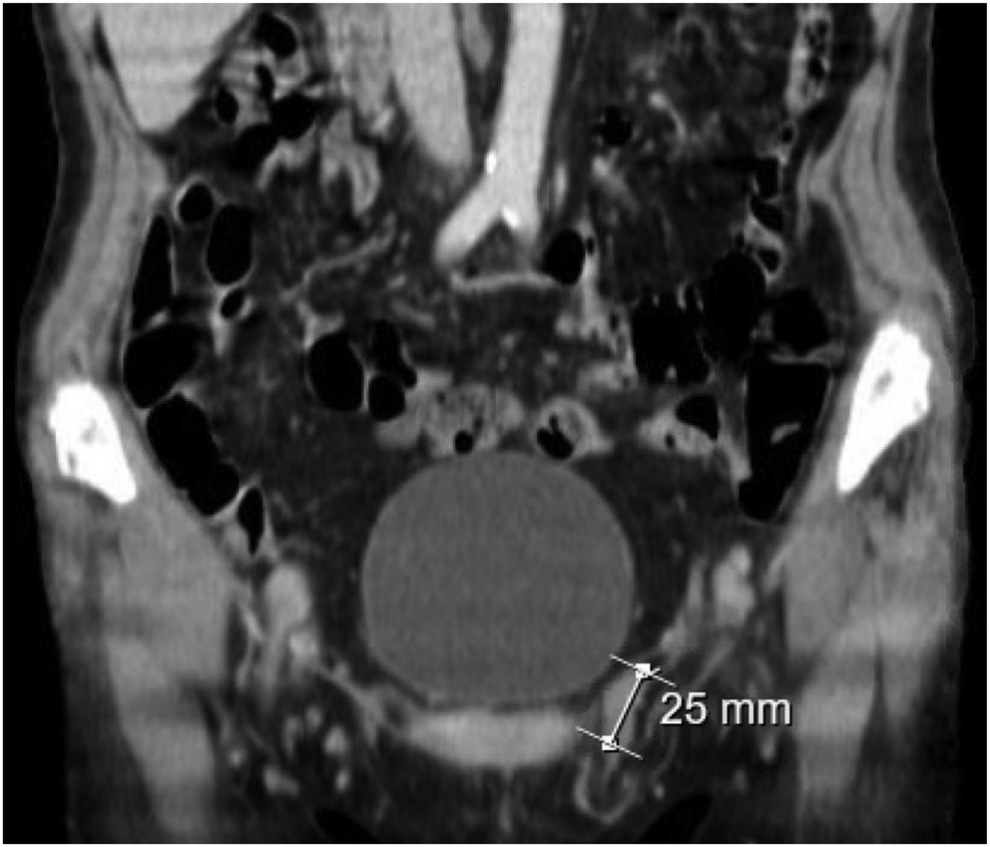
Abdominal CT scan showing a nodular lesion observed in the left inguinal canal with 25 × 10 mm, compatible with a testicular remnant.

On examination, she presented an eunuchoid habitus. She was 175 cm tall with a weight of 51.2 kg (BMI of 16.7 kg/m^2^), arm span of 185 cm, generalized cutaneous hyperpigmentation, and Tanner stage M1P1 with normal female external genitalia. Further analytical evaluations were performed- luteinizing hormone: 64 mIU/mL (NR during follicular phase: 1.8–11.8), follicle-stimulating hormone: 97 mIU/mL (NR: 3.03–8.08), estradiol: 17 pg/mL (NR: 21–251), progesterone: 5.2 ng/mL (NR: 0.1–0.3), 17-OHP: 0.19 ng/mL (NR: 0.21–1.45), and total testosterone: 0.03 ng/mL (NR: 0.11–0.56). CT scan revealed suprarenal bilateral hyperplasia (right width 11.5 mm and left width 12.9 mm) and absence of female internal genitalia (Fig. 1). A nodular lesion was observed in the left inguinal canal with 25 × 10 mm, compatible with a testicular remnant (Fig. 2). DEXA osteodensitometry showed osteoporosis of femur neck and lumbar spine (*T* score –3.5 and *T* score –3.7, respectively). A diagnosis of 17OHD was assumed based on the physical examination findings, history of hypertension, analytical results, and imaging findings. A genetic test confirmed the diagnosis by identifying the pathogenic c.3G>A p.(Met1?) variation in homozygosity in the *CYP17A1* gene. The karyotype analysis came out as 46,XY.

## Treatment

After being stabilized, hydrocortisone was switched to dexamethasone 0.75 mg, besides spironolactone 100 mg b.i.d., olmesartan 40 mg b.i.d., and nifedipine 60 mg b.i.d. She was able to be discharged 2 weeks after, with a blood pressure of 140/90 mmHg and normal potassium levels (3.9 mEq/L), with no need for oral supplementation. She had several appointments, needing medication adjustments, first to achieve better blood pressure control, and also due to iatrogenic Cushing, dexamethasone was reduced to 0.5 mg b.i.d.; secondly, due to hyperkalemia, spironolactone was also reduced to 50 mg b.i.d. She was also started on alendronate + cholecalciferol 70 mg + 5600 UI once a week and calcium carbonate 1250 mg b.i.d. The patient was referred to the urology team for gonadectomy.

## Outcome and follow-up

At the last visit in May 2022, the patient was on dexamethasone 0.5 mg b.i.d., spironolactone 50 mg b.i.d., olmesartan 40 mg b.i.d., nifedipine 60 mg b.i.d., and nebivolol 5 mg b.i.d., added to further control hypertension, with blood pressure recorded as 140/80 mmHg. The analytical study showed ACTH 135 pg/mL, K+ 3.8 mEq/L, and plasmatic aldosterone 2.52 ng/dL.

## Discussion

Phenotype severity of 17OHD depends on the resultant enzymatic activity from the mutation in the *CYP17A1* gene. Presently, there are more than 100 different mutations identified, although phenotype variation has been described for the same mutation (2, 12, 13, 14, 15). The clinical presentation of the patient we report coincides with the classic presentation of 17OHD, which includes hypertension, hypokalemia, and the absence of secondary sexual characteristics (16). Individuals with the 46,XY karyotype usually have a female phenotype with infantilism or ambiguous external genitalia, correlating with the severity of the enzymatic blockage, and it has been shown that more than 25% of normal activity is necessary for normal fetal masculinization; in contrast, 46,XX individuals have a female phenotype with infantilism (12, 17).

In CAH, a low level of cortisol causes ACTH hyperproduction and secretion, resulting in adrenocortical hyperplasia; in 17OHD, accumulation of progesterone and pregnenolone occurs, as well as overproduction of 17-desoxycorticosteroids like deoxycorticosterone and corticosterone (11) – these were not determined in our patient because they were not available in our hospital. However, progesterone levels (which is a substrate of 17-alpha hydroxylase) were found increased in our patient. Data reported in the literature indicate that most patients with this disease have low or normal aldosterone levels; however, like our patient, there are reports of patients with high aldosterone levels (7, 12, 18). The explanation for the decrease in aldosterone levels lies in the inhibitory effect of desoxycorticosterone, which can suppress the renin–angiotensin–aldosterone axis by increasing sodium reabsorption and increasing volume (7). It is thought that in patients with increased aldosterone levels, this is associated with a more severe deficit of 17OH, with increased action of corticosterone methyl oxidase on fasciculate cells, which will lead to an increased aldosterone production from corticosterone, which may have some characteristics compatible with glucocorticoid-remediable hyperaldosteronism (19). This hypothesis may be the presumptive explanation for our patient.

Although the renin–angiotensin–aldosterone axis is independent of the hypothalamus–pituitary–adrenal axis, the mechanism for low renin seems to be explained by the elevated level of aldosterone, an indirect effect of ACTH stimulation, which ultimately downregulates renin production. In this patient, rhabdomyolysis and changes in cardiac electrical activity were probably due to severe hypokalemia, which was described in another case reported in the literature (5). For patients with the 46,XX karyotype, secondary amenorrhea can occur if there is enough enzyme activity for the production of estrogens, which can be achieved with only 5% of regular enzyme activity, as reported in another clinical report (13). In 46,XY individuals, secretion of the anti-Mullerian hormone at the embryonic stage leads to regression of the Mullerian ducts; the posterior absence of androgens generates hypoplastic testes, whose location may vary (intra-abdominal, inguinal, or in the labioscrotal fold), resulting in female or ambiguous external genitalia. Prophylactic gonadectomy must always be performed due to the risk for malignancy (13, 20, 21). Based on physical examination findings and on the results of the analytical assessment, clinical suspicion of 17OHD was considered as highly probable, and diagnosis was confirmed by genetic testing. The described mutation has already been identified and occurs in the initiation codon; so it is predictable that a non-functional protein is generated. In addition, this variant has already been reported, as well as others affecting the same residue (22).

The treatment of 17OHD comprises the administration of glucocorticoids, inhibiting the production of ACTH and, consequently, the synthesis of desoxycorticoids. However, in this patient, the use of glucocorticoids and mineralocorticoid antagonists was insufficient to obtain blood pressure control. When a diagnosis is made later in life, prolonged hypertension may render this therapy insufficient (7, 23). The use of mineralocorticoid antagonists, such as spironolactone, and calcium channel blockers, such as nifedipine, has been used to achieve better blood pressure control (7, 23). In this patient, spironolactone 100 mg bid was added but hyperkalemia was developed, needing to reduce that daily dosage to an half and starting a calcium channel blocker, a angiotensin II receptor blocker, and a beta-blocker. Regarding estrogen replacement, it was the patient's option not to undergo therapy; however, given the patient's age, taking into account an ideal age for menopause between 50 and 55 years and the potential improvement of the cardiometabolic profile and bone health, it would have been preferable (24).

Despite the diagnosis of 17OHD at a late age, this situation is not unique, as another case of a 66-year-old patient has already been described (24).

In conclusion, 17OHD is a rare disease that should be considered in adult patients who present with hypertension, hypokalemia, and lack of development of secondary sexual characteristics.

## Declaration of interest

The authors have no conflicts of interest to declare.

## Funding

This research did not receive any specific grant from any funding agency in the public, commercial, or not-for-profit sector.

## Patient consent

Written informed consent for the publication of her clinical details and clinical images was obtained from the patient.

## Author contribution statement

B Bouça – patient follow-up, elaboration of the manuscript. M Cascão, P Fiúza, S Amaral, P Bogaho – patient follow-up, revision of the manuscript. Silva-Nunes – revision of the manuscript.

## References

[1] Bao X Ding H Xu Y Cui G He Y Yu X & Wang DW. Prevalence of common mutations in the CYP17A1 gene in Chinese Han population. Clinica Chimica Acta; International Journal of Clinical Chemistry 2011 412 1240–1243. (10.1016/j.cca.2011.03.019) 21420394

[2] Auchus RJ. Steroid 17-hydroxylase and 17,20-lyase deficiencies, genetic and pharmacologic. Journal of Steroid Biochemistry and Molecular Biology 2017 165 71–78. (10.1016/j.jsbmb.2016.02.002) 26862015 PMC4976049

[3] DeVore NM & Scott EE. Structures of cytochrome P450 17A1 with prostate cancer drugs abiraterone and TOK-001. Nature 2012 482 116–119. (10.1038/nature10743) 22266943 PMC3271139

[4] Wong SL Shu SG & Tsai CR. Seventeen alpha-hydroxylase deficiency. Journal of the Formosan Medical Association 2006 105 177–181. (10.1016/S0929-6646(0960342-9) 16477341

[5] Philip J Anjali Thomas N Rajaratnam S & Seshadri MS. 17-Alpha hydroxylase deficiency: an unusual cause of secondary amenorrhoea. Australian and New Zealand Journal of Obstetrics and Gynaecology 2004 44 477–478. (10.1111/j.1479-828X.2004.00275.x) 15387879

[6] Kim SM & Rhee JH. A case of 17 alpha-hydroxylase deficiency. Clinical and Experimental Reproductive Medicine 2015 42 72–76. (10.5653/cerm.2015.42.2.72) 26161337 PMC4496435

[7] Kater CE & Biglieri EG. Disorders of steroid 17 alpha-hydroxylase deficiency. Endocrinology and Metabolism Clinics of North America 1994 23 341–357. (10.1016/S0889-8529(1830101-4) 8070426

[8] Moreira AC Leal AM & Castro M. Characterization of adrenocorticotropin secretion in a patient with 17 alpha-hydroxylase deficiency. Journal of Clinical Endocrinology and Metabolism 1990 71 86–91. (10.1210/jcem-71-1-86) 2164530

[9] Moreira AC Leal AM & Castro M. Adrenocorticotropin-corticosterone relationship during dexamethasone therapy in 17 alpha-hydroxylase deficiency. Hormone and Metabolic Research = Hormon- und Stoffwechselforschung = Hormones et Metabolisme 1992 24 339–341. (10.1055/s-2007-1003328) 1325404

[10] Miller WL. The syndrome of 17,20 lyase deficiency. Journal of Clinical Endocrinology and Metabolism 2012 97 59–67. (10.1210/jc.2011-2161) 22072737 PMC3251937

[11] Larson A Nokoff NJ & Travers S. Disorders of sex development: clinically relevant genes involved in gonadal differentiation. Discovery Medicine 2012 14 301–309. 23200061

[12] Yanase T Simpson ER & Waterman MR. 17 alpha-hydroxylase/17,20-lyase deficiency: from clinical investigation to molecular definition. Endocrine Reviews 1991 12 91–108. (10.1210/edrv-12-1-91) 2026124

[13] Çamtosun E Şıklar Z Ceylaner S Kocaay P & Berberoğlu M. Delayed diagnosis of a 17-hydroxylase/17,20-lyase deficient patient presenting as a 46,XY female: a low normal potassium level can be an alerting diagnostic sign. Journal of Clinical Research in Pediatric Endocrinology 2017 9 163–167. (10.4274/jcrpe.3839) 28008861 PMC5463290

[14] Brooke AM Taylor NF Shepherd JH Gore ME Ahmad T Lin L Rumsby G Papari-Zareei M Auchus RJ Achermann JC, A novel point mutation in P450c17 (CYP17) causing combined 17alpha-hydroxylase/17,20-lyase deficiency. Journal of Clinical Endocrinology and Metabolism 2006 91 2428–2431. (10.1210/jc.2005-2653) 16569739

[15] Keskin M Uğurlu AK Savaş-Erdeve Ş Sağsak E Akyüz SG Çetinkaya S & Aycan Z 17α-Hydroylase/17,20-lyase deficiency related to P.Y27*(c.81C>A) mutation in CYP17A1 gene. Journal of Pediatric Endocrinology & Metabolism 2015 28 919–921. (10.1515/jpem-2014-0444) 25719302

[16] Biglieri EG Herron MA & Brust N. 17-hydroxylation hydroxylation deficiency in man. Journal of Clinical Investigation 1966 45 1946–1954. (10.1172/jci105499) 4288776 PMC292880

[17] New MI. Male pseudohermaphroditism due to 17 alpha-hydroxylase deficiency. Journal of Clinical Investigation 1970 49 1930–1941. (10.1172/JCI106412) 5456802 PMC322683

[18] Cottrell DA Bello FA & Falko JM. 17 alpha-hydroxylase deficiency masquerading as primary hyperaldosteronism. American Journal of the Medical Sciences 1990 300 380–382. (10.1097/00000441-199012000-00007) 2264576

[19] Yamakita N Murase H Yasuda K Noritake N Mercado-Asis LB & Miura K. Possible hyperaldosteronism and discrepancy in enzyme activity deficiency in adrenal and gonadal glands in Japanese patients with 17 alpha-hydroxylase deficiency. Endocrinologia Japonica 1989 36 515–536. (10.1507/endocrj1954.36.515) 2555148

[20] Jiang JF Deng Y Xue W Wang YF Tian QJ & Sun AJ. Surgical Therapy of 17α-hydroxylase Deficiency in 30 Patients. Zhongguo Yi Xue Ke Xue Yuan Xue Bao. Acta Academiae Medicinae Sinicae 2016 38 559–562. (10.3881/j.issn.1000-503X.2016.05.012) 27825414

[21] Martin RM Lin CJ Costa EM de Oliveira ML Carrilho A Villar H Longui CA & Mendonca BB. P450c17 deficiency in Brazilian patients: biochemical diagnosis through progesterone levels confirmed by CYP17 genotyping. Journal of Clinical Endocrinology and Metabolism 2003 88 5739–5746. (10.1210/jc.2003-030988) 14671162

[22] Wang W Han R Yang Z Zheng S Li H Wan Z Qi Y Sun S Ye L & Ning G. Targeted gene panel sequencing for molecular diagnosis of congenital adrenal hyperplasia. Journal of Steroid Biochemistry and Molecular Biology 2021 211 105899. (10.1016/j.jsbmb.2021.105899) 33864926

[23] Mantero F Opocher G Rocco S Carpenè G & Armanini D. Long-term treatment of mineralocorticoid excess syndromes. Steroids 1995 60 81–86. (10.1016/0039-128x(9400018-8) 7792822

[24] Guenego A Morel Y Ionesco O Mallet D & Priou-Guesdon M. A late 17α-hydroxylase deficiency diagnosis that leads to the discovery of a new CYP17 gene mutation. Annales d'Endocrinologie 2015 76 71–74. (10.1016/j.ando.2014.11.003) 25613935

